# A Treatise on Hysteria

**Published:** 1830-07-01

**Authors:** 


A TREATISE ON HYSTERIA. By *George Tate*, Member of the Royal College of Surgeons. 8vo. pp. 134. Highley, 1830.

An ignis fatuus that bewitches,
And leads men into pools and ditches.

HYSTERIA has certainly bewildered the practitioner as much as any disease in the extensive catalogue of human infirmities ;—but as it is not considered dangerous, and as it assumes so many different shapes as to be almost indescribable, it has not hitherto obtained the honour of monography in the English language. Mr. Tate's endeavour to draw attention to a malady, which is a kind of epitome or imitator of all the other diseases to which flesh is heir, deserves, and will, no doubt, receive, the serious consideration of his brethren. The following quotation from the opening of the second chapter, will probably be objected to, as simplifying too far the etiology of hysteria.

"With the exception of those cases, real or affected, which are so frequently occurring in what have been called 'the refined circles,' occasioned sometimes by sudden impulse, and sometimes by mere caprice, Hysteria, in all its varieties, whether it be mild, yielding to a brisk cathartic potion,—whether it be of another form, lasting for weeks,—or whether it be more obstinate, persisting for months, or even years,—has one common cause which is essential to its appearance ; namely,—an irregular or defective menstruation. Since I have been attentive to cases of Hysteria, I have never seen one, (with the above unimportant exceptions) either of a simple or of a complex character, in which there did not co-exist distinct traces of a faulty menstruation. There is always some deficiency or some depravity of this secretion : it will be found sometimes altogether suspended ; sometimes redundant, or too frequent in its recurrence ; sometimes dark and grumous ; at others, pale and watery ; sometimes it is attended with agonizing pain and sickness. Sometimes, also, Hysteria will take place previously to, and be indicative of, the first appearance of the menses; and sometimes it will occur when these are about to be no more seen. The common conditions, however, under which Hysteria prevails, are catamenial suppression, insufficiency, or depravity." 12.

The author will not positively deny that hysteria does not take place in men; but he has never seen such a case, and reasoning induces him to doubt its existence. He thinks it probable that such instances as are on record, were cases of chorea, and not of hysteria.

"Debility produced by, or at least combined with, a deranged state of the stomach, liver, and bowels, certainly predisposes to Hysteria ; and delicate females, who are easily excited, are more susceptible of it than the robust ; but there is still something wanting to account for the singular phenomena that this affection exhibits. These phenomena are different from those presented by any other disease : they are perpetually changing their character,—adopting the image of the most terrific maladies, — and are scarcely ever seen in two cases precisely alike. The cases to be afterwards adduced, will, I think, prove conclusively, that defective menstruation is solely accountable for all these manifestations, whatever may have occasioned that function to be deranged. I assume this, because, in the first place, Hysteria is not confined to women of a delicate texture, but sometimes attacks the most hardy and the most healthy ; and, secondly, because a suppressed or disordered uterine secretion is always the forerunner of it, in whatever shape it presents itself." 14.

In the third chapter, Mr. Tate divides hysteria into three degrees of intensity. The first or mildest degree occurs almost invariably between the ages of 13 and 45—is always accompanied by some irregularity of the menstrual discharge—and often by disorder of the digestive organs. The characteristics are well known—alternate fits of weeping and laughing—starting and screaming—death-like stillness and gigantic struggles—clangor intestinorum, globus hystericus—pale urine, the latter not constant. For the cure of this degree of hysteria, the most nauseous drugs selected from the three kingdoms of Nature, have been freely administered, and "as hysterics are occasionally brought on by passions of the mind, the patient had only to make her election, either to exercise at once a becoming control over herself, or to indulge her sensibility at the expense of being drenched with the most suffocating liquids in the world, and of having her convulsions of caprice exchanged for convulsions of disgust." In such cases, the penalties may (he thinks) be well incurred, and may perhaps tend to induce susceptible young ladies to divest themselves of fanciful illness. We are not, however, quite so well satisfied as Mr. Tate seems to be, that even this first grade of hysteria, is ever actually *fanciful*. If the corporeal disorder arise from mental emotions, it is as real while it last, though not so difficult to conquer, as when resulting from uterine irregularity. But as Mr. Tate admits that this first grade of hysteria, exhibiting, indeed, its simplest form, may "arise from some mental emotion, where there is clearly *nothing wrong in the animal functions*," the admission is fatal to his etiological conclusion, that hysteria is essentially dependent on "irregular or defective menstruation."

If a mental cause can produce the disease in one degree of intensity. it will be difficult to persuade the profession that the same cause may not be adequate to the production of a higher grade of the malady. Mr. T. protests against the utility of the foregoing remedies in the more common forms and degrees of hysteria, where the disorder is connected with a corporeal malady.

"The first object, in the treatment of this form of disorder, is to cleanse the bowels; and this is most effectively done by a brisk cathartic of calomel and jalap, followed by castor oil. In a great majority of cases, a brisk action upon the bowels will be attended with immediate relief of the fits or paroxysms, or whatever else they may be called, and they will rarely return if the subsequent practice be judicious: which consists merely in avoiding stimulants; in living on a bland and nutritive diet, and taking aloes and iron with some aromatic oil, until the uterine and alvine secretings are properly regulated. It has frequently happened, in the course of the few years that I have been in practice, that after having relieved a young female from the immediate attack, I have represented to her mother the necessity of repairing the deranged state of her general health; and those girls who have been for years deprived of their natural health, going about with sallow and sickly faces, parched and pallid lips, furred tongues, and limbs incapable of the least exertion, have been indebted to a few doses of calomel and jalap, followed by pills of aloes and iron, for the perfect re-establishment of their strength, health, and beauty." 19.

This treatment, however, will only apply to the first degree of hysteria occasioned by "over-excitement of mind and nothing more"—but where the fits recur frequently, and where "the general health and uterine secretion are found to be deranged—the same treatment is not only useless and unreasonable—it is positively mischievous."

### HYSTERIA OF THE SECOND DEGREE.

This is of much more serious consequence than the former. It generally arises suddenly, with some singular and unaccountable symptom, very alarming to the patient's friends, and occasioning the sudden summons of the medical attendant. If he be not on his guard, he will be very apt "to mistake this disorder for some real disease or some active internal inflammation. He may thus do more mischief than all his subsequent treatment can repair." We shall abbreviate a case or two in illustration.

*Case* 1. A. W. aged 19 years, a rosy-cheeked healthy-looking girl complained, on the 22d of April, of violent pain in her eyes, which seemed inflamed, and discharged a copious flow of scalding tears, with extreme intolerance of light. This had come on without previous shivering or other warning, a few hours previously.

"The conjunctiva was about as much injected as it is generally after a violent fit of crying. She was immediately bled from the arm; and after losing about eight ounces of blood, she opened her eyes, and declared she could see as well, and bear as much light as ever she could in her life. The pain, also, was nearly gone; and this without any fainting or any perceptible tendency to it. She was then ordered to go home, to keep quiet, and to live low for a day or two; calomel and jalap, with sulphate of magnesia, were also prescribed for her. At about four o'clock on the following morning, I was called up to go to her immediately, (six miles into the country) as the people about her declared she must die, unless she could obtain instant relief. I found her seemingly in agonies. Her eyes continued well; but she was breathing with such excessive rapidity as I can only compare with that of a hound after a hard run, and with much the same kind of muscular distress. Her hand was pressed firmly against her left side, beneath the breast, where her gestures (for she could not speak) signified that she was suffering acute pain. It was impossible to ascertain the state of her pulse, in consequence of the agitated state of the respiratory system, to say nothing of her terror; but her chest sounded well, and she was in a profuse perspiration, attended with high heat of the whole surface of the body. Upon inquiry, I found that she had not menstruated for fourteen weeks, and for more than twelve months very inadequately to her former habits; and had complained of pain in her left side, with occasional palpitations. These circumstances shed some light upon the rather puzzling appearances of the case, and went a great way to determine its real source and character. I then had her turned round, to get an examination of the spinal column. On making pressure upon the four uppermost dorsal vertebræ, she complained of great tenderness, and pain; which was referred to the left side, and to the scrobiculus cordis. As I had always found these, or some other divisions of the spine, tender, on the application of pressure, in urgent cases of Hysteria, I was quite satisfied that this was nothing more than a mysterious case of that description. The fugitive nature of the apparent ophthalmia, the seat and kind of pain in the left side, the pain in the dorsal vertebræ, with a suspended menstruation; all concurred in giving it this and no other character. Although not expecting much benefit from it, at the solicitation of friends, she was again bled, with scarcely any relief. The treatment which I chiefly relied upon was the tartar emetic ointment to the spine. This was applied along the whole course of the dorsal vertebræ, three times a day; and she took calomel and cathartic extract, followed by an aloetic mixture, every four hours." 24.

Dark and offensive evacuations followed, and the pain and quick breathing were relieved. When the tartar-emetic had produced a copious crop of pustules the other symptoms gave way, and she gradually recovered health and strength under the use of aloetic medicines with steel. But the catamenia did not appear, and at the end of six weeks, she was again attacked in a precisely similar manner. The tartar-emetic was re-applied, and again she recovered. Under the use of aloetics she at length menstruated, and there was an end of the business. The next case we shall give in the author's own words:

*Case* 2. "Miss W., aged 15, was taken ill at a boarding school, in April, 1826. For a few days she had complained of head-ache and loss of appetite; and without any further warning, awoke on Sunday morning, after a tranquil night, with a train of symptoms resembling Tetanus. Her governess sent to me in great alarm. The following was pretty nearly her condition when I first saw her: she was lying upon her head and her heels, her back being thrown into an arch, and scarcely touching the bed-clothes. Her arms were flexed and rotated inwards; her fingers violently closed, grasping her thumbs, which were stuck into the palms, in a way that is frequently seen in hydrocephalic children. Her toes were bent inwards, and her legs bent and twisted in the same manner as her arms. It was with great difficulty that her hand could be forced open, although the attempt did not much annoy her. She was perfectly sensible, and complained of violent heat and pain in the head. Intolerance of light was very great; and when her eyelids were opened, she squinted frightfully. Her respiration was short, and she complained of pain in the side, and palpitation. Her pulse were 110; her tongue clean; skin hot, but covered with moisture; she was thirsty, and said her mouth was dry. Her general health had been previously good. *She had never menstruated.* Such was the striking appearance of the case; which, from the suddenness of the attack, after passing a good night, and from her having never menstruated, with the corresponding symptoms, I strongly suspected was nothing more than a strange form of Hysteria. Under this impression, I examined the spine, and the moment pressure was applied between the scapulæ, upon the upper dorsal vertebræ, the patient complained of pain, which was also manifested in the shrinking expression of her countenance. That which was conjecture before, thus became matter of certainty, and I felt myself warranted in assuring the governess, who was naturally in considerable alarm, that these formidable symptoms were mere phantoms, which would readily disappear; and that a few days would, probably, be sufficient to restore her to her usual good health. The infrication of the tartar emetic was immediately begun throughout the dorsal region; and calomel and jalap were prescribed for her. As soon as the bowels were freely evacuated, her head was better, and respiration was relieved; but the spasmodic, or rather, tetanic affection, did not yield at all. In about thirty-six hours, the antimonial ointment had accomplished its duty; when the spasm was immediately influenced, the flexors gradually relaxed, and, in less than twenty-four hours after the pustulation was developed, not a vestige of the disorder remained. The contractions returned twice or thrice, to a partial extent, in the course of the following month; sometimes one thumb, and at another time one or two fingers, being bound down; and, upon one occasion, this lasted for several days; when a second application of the ointment was, very reluctantly, consented to. She had, afterwards, no return of pain or disorder. During the whole of this time, aloetics, with iron, were daily administered; and, at the expiration of five weeks from the accession of her illness, she menstruated; and was afterwards quite well. Thus proving, very satisfactorily, that the amenorrhœa was the source of the vertebral irritation; and that this, in its turn, produced the other ailments." 29.

*Case* 3. This was also a young lady, who had been out of health for four years. When first observed by Mr. Tate, she had acute pain in the left side, increased on inspiration but relieved by pressure. She also complained of pain in her head and oppression about the chest. She would occasionally fall down apparently lifeless, and lie so for half an hour, recovering at intervals and then speaking rationally. When seemingly comatose, her breathing would be suspended for ten minutes or longer at a time, or carried on with such subtleness that no air escaped her lips. Then a rapid gasping would follow, to be succeeded by another death-like stillness, &c. All this time the pulse was quiet and regular. She intreated Mr. T. to bleed her, which he did, suspecting that there was some disease of the heart; but he soon saw the case in its true colours. Having convinced himself that there was no disease of the head or thoracic organs, he proceeded to examine the spine, where he found uneasiness complained of when pressure was made on the dorsal region. "Upon increasing the pressure, the pain was increased, and passed through to the pit of the stomach and to the left side, at the spot so complained of—causing the breathing to be oppressed." The catamenia were found to be unusually scanty and dingy. The antimonial ointment was applied to the spine, and as soon as the eruption came out the relief was astonishing. "The fits went off, the head and side were no longer complained of, and the palpitation gradually subsided."

*Case* 4. "Elizabeth M., aged 20. Early one morning, I was sent for to this young woman, and found her in bed, where seven or eight persons were employed in keeping her by main force. She had complained for some days of a bad head-ache; was of a pale, delicate complexion, of a very slender frame, and had been for many months without any uterine evacuation. She had waked in the night, screaming out like a maniac, to the terror of all the family; and, in attempting to get out of bed, had fallen back in a state of insensibility, and had continued so up to the time of my arrival. She was struggling with amazing violence; her eyes were staring wildly—she was grinding her teeth,—her hands clenched, and every muscle of the body seemed to be thrown into a state of most tremendous spasm. This was Hysteria, clearly enough. So far there was little difficulty in deciding. Her pulse being rapid and bounding, some blood was drawn, but without affording her the smallest relief. Calomel and jalap were, with some difficulty, forced into the stomach. When these had copiously relieved the bowels, she became calm, and the convulsive throes ceased; but the insensibility was unabated, and she lay like a girl perfectly dead, till the middle of the following day. I had already begun the tartar emetic inunction, and when she was sufficiently sensible to answer, I traced the course of the spine, and she complained and shrunk away when the fingers were applied upon the dorsal vertebræ. The pain was felt through the whole chest, particularly at a spot beneath the left breast. Indeed, I have scarcely met with a case in which the spinal affection was more strongly and clearly marked. Besides the tenderness of the spine, and the pain in the left side, there was, in this case, excessive tenderness in the right side, under the margin of the ribs; this was so great, that she dreaded the slightest manual examination, even before she was touched. The pain was confined to the hepatic region, but was too acute and too superficial to induce a suspicion that it was connected with visceral disease. It was, as well as that of the other side, occasioned by the spinal disorder; and as soon as this was relieved by the usual application of the ointment, and the menses were restored by the usual combination of iron and aloes these pains were dispersed, and the young woman afterwards acquired greater strength and better general health, than she remembers to have enjoyed at any former period of her life. This, as I before remarked, has been the usual result of Hysterical cases, treated in the manner above described." 35.

The symptoms of hysteria of the second degree are thus adverted to. *First*, there is defective menstruation. This our author considers as the "head and front of the case"—the original cause of the disorder. The *next* circumstance, "and the most important of the whole list," is "distinct pain upon the application of pressure or of heat, to three or four of the six superior dorsal vertebræ." Upon this point our author desires to fix the attention of his readers—"for this spinal affection, whatever its intrinsic quality, is clearly chargeable with most of the curious images, and fantastic forms, that Hysteria is accustomed to put on; and yet, notwithstanding its constant occurrence in these forms of Hysteria, and its frequent existence where there is even a *tendency* to Hysteric disorder, it is a circumstance that has been overlooked by those who have professed to treat upon the subject, as well as by those who, for the sake of gratifying curiosity, have published detached cases of Hysteria under various other designations." In other parts of the spine, especially in the lumbar vertebræ, pain is frequently complained of; but in the dorsal region no uneasiness is usually felt till pressure be made. Proceeding downwards along the spine, the patient shrinks when we reach the dorsal vertebræ, and she acknowledges the existence of pain, which sometimes, not always, shoots through to the chest or to the left side, sometimes to both, generally oppressing the breath. Mr. Tate does not attempt to account for this curious phenomenon. He contents himself with stating the fact, leaving others to unravel its philosophy. Fortunately, or unfortunately, no opportunity of examining the spine, *post mortem*, has occurred among his hysterical patients; and, therefore, the precise pathological condition of the part must be matter of conjecture.

The pain in the left side is next to be attended to.

"It is usually situated immediately below the left breast, in a hollow formed between the cartilages of the fifth and sixth, or sixth and seventh ribs; it is generally so circumscribed, that it may be covered by a shilling; and is of the gnawing kind. Occasionally, however, it is most acute, feeling as if a knife were being stuck into the spot, and the patient cannot forbear screaming. This pain is complained of for some time before the invasion of the Hysteria. The patient is often observed to incline the upper part of the body to that side, dropping the left shoulder, which relaxes the painful part and affords some relief. The act of raising the left arm above the head, or of bringing the body into a perfectly erect position, is attended with an increase of pain. I apprehend this pain is really seated in the intercostal nerve, although I have sometimes thought it must be situated in the nerves of the heart itself; as it is difficult to account for its perpetual preference for the left side. The right side, certainly, is often not exempt from pain; but, in nineteen cases out of twenty, the prominent grievance is in the former; and in the like proportion of instances, I can put a finger on the spot with as much certainty as if it were visibly marked." 43.

Mr. T. is convinced that many spinal curvatures have arisen in consequence of this pain, causing a tendency to lean the body constantly towards the affected side.

Palpitation is another symptom which is almost always present. So is pain in some part of the head, generally in the front or occiput, or both. Globus hystericus though not always present, is very often so.

### HYSTERIA OF THE THIRD DEGREE.

It has been stated that the various forms of this kind or degree of hysteria, appear to be caused *immediately* by the spinal affection, which, in its turn, is the result of some occult association or sympathy between the spinal cord and the uterus. We have, therefore, two indications to fulfil—the removal of the immediate cause of the hysterical evolutions; and the restoration of uterine vigour and health. The modes of accomplishing these objects are fully pointed out in the cases above detailed.

"In some instances, where the patient is very robust,—the cheeks highly flushed,—the eye injected,—the forehead red and polished, it may be useful to abstract blood by the lancet; but it rarely does much good, and, as far as I have seen, never relieves the immediate attack. But when symptoms so sudden and alarming make their appearance, a medical man is expected to do something *instanter;* and in strong young women, bleeding does no harm. In delicate girls, on the contrary, it aggravates the disease tenfold; and renders the cure infinitely more difficult and tedious than it would otherwise be. As a general rule, therefore, venesection should not be performed, without some very substantial reason. It neither removes the pains, nor the spasms; but very often prolongs both." 47.

Having made a careful examination of the spine, and ascertained the seat of pain, the first thing to be done is the application of the tartar-emetic, either by friction or plaster on the spot. If the symptoms be urgent, whether cataleptic, choreiform, tetanic, hemiplegiac, or Proteian, the application should be carried along the whole course of the vertebræ—and this should be done every six or seven hours, until the pustulation is fully developed. "The hysterical symptoms will then yield, and the patient will become calm and sensible." But, as the cause of the spinal affection, the uterine disorder, is still in operation, it is sometimes necessary to establish the eruption, *iterum iterumque*, in order to secure the patient from relapse.

Leeching has always failed to relieve the spinal tenderness in our author's practice, and the same may be said of blistering. Purgatives are necessary, as auxiliaries. The evacuations are generally black and unhealthy at first.

"We have thus disposed of the immediate attack; but another important indication in the treatment remains to be considered. This is to re-establish a healthy and vigorous menstruation. Now the mal-performance of this important function may be of several different kinds; it may consist in absolute suspension or suppression; in being before or beyond the usual period; in being of a dark and grumous, or of a very pale complexion; in being too copious or too small in quantity; or in being attended with excruciating pain. Such being the various states of disorder, it will be seen how impossible it is to lay down any rule of treatment that can be universally applicable. Each must be separately considered, and treated according to the discretion of the practitioner, upon the usual principles; in first of all, improving the secretions, and placing the digestive apparatus in a state of reparation. After this has been effected, wholesome air, wholesome food, and wholesome exercise, with preparations of iron, and the use of the warm, tepid, cold, or shower bath, according to the circumstances, will generally be the best tonics, and restore the patient to her usual health and strength." 51.

Mr. Tate next introduces a number of cases, chiefly from the MEDICO-CHIRURGICAL REVIEW, where the patients evidently laboured under hysteria, but where the diseases were designated by various appellations, according to the fancy or judgment of the practitioner. The criticisms on these cases are judicious and candid. The perusal of them will be advantageous to the reader.

With the following graphic sketch of the symptomatology of this third degree of hysteria, we shall close this section.

"As there is no regular set of symptoms that admit, as in common diseases, of being set forth as universally present to mark its nature, I must content myself with a general description of this form of Hysteria, leaving the body of the picture to be filled up by a report of cases. As before stated, in the most tedious form of Hysteria, menstruation is always more or less faulty at the onset, and as the case advances, this becomes suppressed altogether, or is performed very sparingly, perhaps only once in many months, and then with great pain. Where this function is quite suspended, there is, generally, neither any periodical pain, nor any sensation, to show that nature had not forgotten this customary duty. Shortly afterwards, the patient becomes weak and desponding, loses her appetite, and the bloom from her cheeks. She has still nothing particular to complain of, and, generally, keeps up her flesh, although it has every appearance of relaxation. If a medical man sees her now, he will find her with a moist and tremulous tongue; being foul at the root, and having the papillæ, at that part, larger than natural, and like little tubercles; with a tainted breath; depraved taste; little or no appetite; with a weak, languid pulse; with a sickly, yellowish complexion; black or clay-coloured alvine secretions, and the urine highly coloured and scanty. In a little time, she will have pain under the left breast; which is increased by deep inspiration, and by reclining upon that side,—sometimes pain also in the right side, palpitations, flutterings, sinkings, and, together with these, there will be pain upon pressure in one or more parts of the spine: first of all, in three or four of the dorsal vertebræ; generally, also, in the lumbar, and, if the case be very lasting, it sometimes extends up to the very summit of the cervical portion. In such cases, the head-aches are intolerable; being in some instances constant, in others interrupted, but always violent. The pain is often continued down the arms and into the legs; the extremities are generally clammy and cold.

"In the midst of all this, the patient is not much reduced in flesh, and, for a considerable time, it is not sensibly diminished. As the disease advances, a number of anomalous pains of a neuralgic character, become associated with the other symptoms. Thus, if pressure be made upon the supra or infra-orbital nerves, upon the inferior maxillary, &c. as they issue from their foramina, considerable pain is produced, but I never found these spots complained of, in the absence of such pressure. It is not, however, the facial nerves that are alone implicated, for almost every nerve in the body becomes, at the same time, endued with a similar increase of sensibility. This sort of neuralgic affection is seldom observed until the case is far advanced, and has become equally inveterate and puzzling.

"A condition of this kind will frequently prevail for eighteen months, before any particular notice is taken of it by the patient, or her friends: she gets gradually worse, until some sudden spasmodic affection, or other unaccountable symptom, commands attention; and then medical advice is obtained. At other times, where more solicitude is felt, earlier application is made to the followers of the healing art; and the patient is called a dyspeptic, or a hypochondriac, or a nervous lady; and, if judiciously treated, will gradually recover her health." 93.

A remarkable and protracted case is next detailed, of which we can only give some of the more prominent features in this place.

Miss ——— became our author's patient in March 1825, after ten years' illness, and having been under a pretty long list of learned doctors. She had menstruated at fifteen, and her illness commenced soon afterwards with a total suppression of the catamenia, referred at the time to cold. She was now so weak as to be incapable of walking across the room. Her complexion was sallow—her lips bloodless—pulse small and quick—tongue furred—bowels torpid—dejections various but morbid—urine clear.

"There was a fixed and lancinating pain in a hollow, between the cartilages of the fifth and sixth ribs of the left side; pain under the margin of the ribs of the right side; considerable difficulty of breathing, and frequent violent palpitations. The head-aches were almost incessant, and often nearly distracting by their violence. There was pain upon pressure throughout the cervical and dorsal vertebræ; and pressure between the shoulders, aggravated the dyspnœa. She was sometimes seized with an uncontrollable vomiting, which lasted seven or eight days together; at which times, not a spoonful of cold water would remain upon her stomach; these attacks were ultimately tranquillized by opiate suppositories, leaving her strength completely prostrate. She scarcely ever closed her eyes to sleep, although her sufferings were so great, that she was lying in a recumbent posture, at times for days and nights together, with her eyes shut in silent agony. She appeared literally not to eat anything. *She had not menstruated since the beginning of her illness, when she was near sixteen years of age.*

Slight vexation or surprize threw her into a paroxysm of hysteria. A tartar-emetic plaster was applied to the spine, which occasioned great distress, and as sickness came on about this time, it was placed to the account of the plaster. Mild aperients were given, and she gradually regained a little strength. As she improved, the carbonate of iron was cautiously tried, but brought on the sickness again, and was discontinued. She went to Cheltenham, and a caustic issue was established over the seat of pain in the side, without effect. "After a short interval, the carbonate of iron was again taken, and it now did not appear to offend the stomach. The quantity taken at each dose, was increased, by slow degrees, from a scruple to half an ounce, three times a day; so that, at last, she may be said to have lived upon iron." Under this plan she rallied wonderfully. The pain was relieved—the bowels acted favourably—the head-aches were trifling—and the spinal tenderness scarcely perceptible. The catamenia returned once, and then ceased. She was able to walk and ride. She went to Bath, where a surgeon bled her once a fortnight, for a whole year. She returned to Cheltenham, and presented the following phenomena.

"She had now an irregular pulse—violent palpitations—œdematous legs, even to the knees—cold extremities—shortness of breath—and a countenance indicative of exhaustion and distress. The left breast was very much wasted, as were also some of the muscles on the side of the chest: producing a degree of deformity, that was evident through her clothes. I earnestly entreated her to subject herself to no more such ruinous experiments; but to take wholesome food—to take as much exercise, as her strength would bear, short of fatigue; to take no medicine, but a tonic-aperient pill; and to use the shower bath twice, and the warm hip-bath, three times a week. She then proceeded to Leamington, where she has followed these directions. Her health improves, but the wasting, and numbness, of the left breast and side, are making gradual progress. There appears little hope of her complete recovery, although she has already endured little short of a quarter of an ordinary life of diversified suffering." 99.

Mr. Tate thinks that this must be considered as an instance of prolonged and established disease, "resulting from original error, and continued series of mal-practice." If it be denied that this was a case of hysteria, Mr. T. asks what it was? The case is by no means a solitary one. "Daily are young females, afflicted with this very pain under the left breast, bled, blistered, leeched, cupped, and passed through a long course of depleting and enervating medicines, when there is no earthly necessity for it." We fear this is but too true.

But our limits are far overstepped, and we believe we have shewn samples enough of Mr. Tate's little work to induce the reader to peruse the original. It is really a very meritorious performance, and the coincidence of ideas between Mr. Tate and Dr. Addison, whose books were published on the same day, clearly proves that these ideas are taken from actual observation of facts.

